# 尼古丁诱导肺癌细胞上皮间质转化促进其侵袭转移

**DOI:** 10.3779/j.issn.1009-3419.2016.04.11

**Published:** 2016-04-20

**Authors:** 艳须 侯, 雪冰 李, 振华 潘, 玲玲 祖, 亚光 范, 嘉琮 尤, 玉丽 王, 岷 王, 沛锐 陈, 旺 沈, 清华 周

**Affiliations:** 1 300052 天津，天津市肺癌转移与肿瘤微环境重点实验室，天津市肺癌研究所，天津医科大学总医院 Tianjin Key Laboratory of Lung Cancer Metastasis and Tumor Microenvironment, Tianjin Lung Cancer Institute, Tianjin Medical University General Hospital, Tianjin 300052, China; 2 610041 成都，四川大学华西医院肺癌中心 Lung Cancer Center, West China Hospital, Sichuan University, Chengdu 610041, China

**Keywords:** 肺肿瘤, 尼古丁, 上皮间质转化, 侵袭, Lung neoplasms, Nicotine, Epithelial-mesenchymal transition, Invasion

## Abstract

**背景与目的:**

我们的前期研究发现尼古丁能诱导肺癌细胞上皮间质转化。本研究的目的是探讨尼古丁诱导的上皮间质转化（epithelial-mesenchymal transition, EMT）与肺癌侵袭之间的关系。

**方法:**

应用不同浓度尼古丁处理肺腺癌A549细胞，应用Real-time PCR和Western blot方法检测EMT相关分子标志物E-钙粘蛋白（E-cadherin）和波形蛋白（Vimentin）mRNA和蛋白表达水平，应用免疫荧光技术检测β-链蛋白（β-catenin）蛋白表达位置的变化，应用划痕实验和Transwell小室侵袭实验检测尼古丁对肺癌细胞迁移侵袭能力的影响。

**结果:**

尼古丁明显下调肺癌细胞株A549 E-cadherin mRNA和蛋白水平表达（*P* < 0.01, *P* < 0.01），并具有浓度和时间依赖性；尼古丁明显上调肺癌细胞株A549 Vimentin mRNA和蛋白水平表达（*P* < 0.01, *P* < 0.01）；尼古丁诱导肺癌细胞株A549细胞β-catenin蛋白发生核转移；划痕实验和侵袭实验观察到尼古丁处理的肺癌细胞株A549细胞的迁移和侵袭能力明显增强（*P* < 0.01, *P* < 0.01）。

**结论:**

尼古丁能够诱导肺癌细胞发生EMT，并且促进肺癌细胞株A549细胞的体外侵袭潜能。

## Abstract (en)

**Background and objective:**

Our previous study found that nicotine could induce lung cancer cell epithelial-mesenchymal transition (EMT). The aim of this study is to explore the relationship between nicotine-induced EMT and lung cancer invasion and metastasis.

**Methods:**

Real-time PCR and Western blot were used to detect the expression changes of EMT-related markers, E-cadherin and Vimentin, in A549 lung cancer cells treated with nicotine; The transposition of β-catenin protein expression was determined by immunofluorescence; Scratch test and Transwell invasion assay were used to detect the effects of nicotine on lung cancer cell migration and invasion.

**Results:**

Nicotine can significantly down-regulate the expressional level of E-cadherin mRNA and protein of A549 cells in a manner of dose and time-dependent (*P* < 0.01, *P* < 0.01); Nicotine can significantly up-regulate the expressional level of Vimentin mRNA and protein of A549 cells in a manner of dose and time-dependent (*P* < 0.01, *P* < 0.01); Immunofluorescence results showed that β-catenin protein was significantly transfered to nucleus; Scratch test and Transwell assay showed that Nicotine could remarkably increase the migration and invasion potential of lung cancer cells (*P* < 0.01, *P* < 0.01).

**Conclusion:**

Nicotine can induce cancer cells EMT, and promote the invasion and metastasis ability of lung cancer cells.

肺癌是人类最常见的恶性肿瘤，也是对人群健康和生命威胁最大的肿瘤，并成为癌症相关疾病首要死亡原因^[1-3]^。世界上每年有近两百万人罹患肺癌，约占所有新患癌患者的13%，同时每年又有一百多万人死于肺癌，约占癌症总死亡人数的20%^[2-4]^。美国国家癌症研究所从2004年-2010年登记的数据显示，处于局限期肺癌患者（18%）5年生存率是53.5%，而确诊为晚期的患者（55%）生存率只有3.9%^[5]^。由于肺癌患者早期症状并不典型，多数患者就诊时已处于晚期，导致总体的5年生存率只有约15%^[6]^。80%的肺癌患者为非小细胞肺癌（nonsmall cell lung cancer, NSCLC），吸烟与NSCLC的发生紧密相关，是肺癌的首要相关因素。尼古丁作为香烟中的重要活性成分，可以诱导多种人类癌细胞的上皮间质转化，并促进其侵袭转移^[7]^。

上皮间质转化（epithelial-mesenchymal transition, EMT）是一个高度程序化的过程，细胞间粘连缺失而导致细胞极性消失，并且获得间充质特性，被认为与包括肺癌在内的多种肿瘤细胞的侵袭和转移密切相关^[8, 9]^。肿瘤临床治疗成败的关键在于患者治疗后的复发和远处转移，这是导致死亡的主要原因，约有80%患者的死亡与远处转移有关，因此探讨转移的发生机制对治疗及改善患者预后具有重要的临床意义。E-钙粘蛋白（E-cadherin）和波形蛋白（Vimentin）分别是上皮和间质表型的重要标志物，肺癌细胞发生EMT时，上皮细胞表型表达减少，间质表型则表达增强，造成细胞间连接下降，使得肺癌细胞迁移和侵袭能力增强^[10, 11]^。E-钙粘蛋白作为表皮状态的关键因素，它的部分缺失与多种肿瘤的进展和不良预后紧密相关^[12]^。β-链蛋白（β-catenin）同样也是一种主要存在于细胞膜的粘连蛋白，与E-钙粘蛋白构成复合体，共同组成细胞间粘附分子的重要部分，在抑制肺癌的侵袭转移过程中发挥重要作用^[13]^。已有的研究^[14]^表明：肿瘤细胞膜β-catenin蛋白的减少，常常导致细胞核蛋白积累，这个过程同样伴随着E-cadherin表达减少和Vimentin蛋白表达增多，证明β-catenin蛋白的核转移同样发生了EMT。本研究主要是探讨尼古丁诱导的EMT对肺癌侵袭潜能的影响。

## 1 材料与方法

### 1.1 细胞株与主要试剂

本次实验所使用A549肺癌细胞株由天津市肺癌研究所提供；尼古丁、FITC标记二抗购自美国Sigma公司；DMEM培养基、胎牛血清购自美国Gibco公司；β-actin、E-cadherin和Vimentin单克隆抗体购自美国Cell Signaling公司；β-catenin一抗购自Abcam公司；HRP标记的二抗、细胞蛋白提取试剂购自中国碧云天公司；RNA提取试剂Trizol购自美国Thermo Fisher Scientific公司；M-MLV逆转录试剂盒、SYBR GREEN试剂盒购自日本TAKARA公司；24孔Transwell侵袭小室培养板购自Corning公司。

### 1.2 细胞培养

人肺腺癌A549细胞培养于含10%胎牛血清的DMEM培养基，置于37 ℃、5%CO_2_培养箱中进行培养；每2 d-3 d胰酶消化细胞传代，每1 d-2 d更换1次培养基；取生长良好，处于对数生长期的细胞用于实验，药物处理前一般要先无血清饥饿处理细胞24 h，尼古丁处理浓度分别为0 μmol/L、0.1 μmol/L、1 μmol/L、10 μmol/L。

### 1.3 Western blot

提取尼古丁（0 μmol/L、0.1 μmol/L、1 μmol/L、10 μmol/L）处理后各组细胞蛋白，蛋白样品100 ℃煮沸5 min，使蛋白充分变性，10%SDS-PAGE凝胶电泳分离目的蛋白，并转移至硝酸纤维素膜上；裁剪后的膜用含5%脱脂牛奶封闭液室温封闭2 h，加入稀释后的一抗，4 ℃孵育过夜；次日用TBST缓冲液洗膜3次，HRP标记的二抗室温孵育1 h；再用TBST缓冲液洗膜3次，配置化学发光试剂，孵育2 min，暗室中曝光30 s-5 min，自动洗片机洗片。实验重复3次，应用Imaje软件对目的条带进行灰度分析。

### 1.4 Real-time PCR

应用Trizol法提取处理后各组细胞总RNA，根据TAKARA公司提供说明书进行逆转录和实时定量检测。实验设置三个复孔并至少重复两次。*GAPDH*基因表达量作为内参对照，使用2 ^-ΔΔCt^法行相对定量分析。引物（由华大基因合成）序列如下：GAPDH F: AACCTGCCAAATATGATGAC，R: ATACCAGGAAATGAGCTTGA；E-cadherinF:TGCCCAGAAAATGAAAAAGG，R:GTGTATGTGGCAATGCGTTC；Vimentin F:GGAAATGGCTCGTCACCTTCGT，R:AGAAATCCTGCTCTCCTCGCCT。

### 1.5 免疫荧光实验

取生长状态良好的细胞接种于覆有盖玻片的6孔板，制备细胞爬片，尼古丁处理前细胞饥饿24 h，处理浓度为0 μmol/L和1 μmol/L；继续培养细胞24 h，PBS清洗爬片，-20 ℃条件下无水甲醇固定20 min；0.5%Triton X-100室温通透细胞20 min，5%BSA室温孵育细胞30 min；滴加β-catenin免疫荧光一抗，湿盒内4 ℃孵育过夜；次日FITC-标记二抗室温避光孵育1 h，DAPI复染细胞核；最后抗荧光淬灭剂封片，显微镜玻片观察采集图像并拍照记录。

### 1.6 细胞划痕实验

取生长状态良好的细胞接种于6孔板，待细胞贴壁生长密度达到80%，无血清培养基饥饿处理24 h，使用无菌的200 μL黄枪头垂直六孔板均匀划2条横线，PBS洗涤脱落细胞；添加不同浓度尼古丁（0 μmol/L、0.1 μmol/L、1 μmol/L、10 μmol/L）的培养基继续培养细胞，分别于0 h、24 h和48 h时在倒置光学显微镜下拍摄照片，测量划痕区域面积变化。

### 1.7 细胞侵袭实验

取无血清饥饿处理24 h后的细胞，调整细胞浓度为5×10^5^个/mL，分别用含0 μmol/L和1 μmol/L尼古丁的培养基重悬细胞，将Transwell小室置于24孔培养板内，上室中加入100 μL细胞悬液，下室中加入500 μL含5%胎牛血清的DMEM培养基，置于37 ℃、5%CO_2_培养箱中培养20 h，擦净上室内残余的细胞和基质胶，甲醛室温固定细胞20 min，PBS洗涤3次，结晶紫染色洗净后，显微镜下随机取3个-5个不同视野进行拍照计数，取平均值，计算迁移细胞的相对数目，每组平行设2个复孔，实验重复3次。

### 1.8 统计学方法

实验数据计量资料以Mean±SD表示，应用GraphPad Prism 5统计软件对进行统计学分析和绘图；两组间比较采用*t*检验，多组间比较采用单因素方差分析，两两比较时采用LSD检验法或Dunnett-t法。双侧界值*P* < 0.05为差异有统计学意义。

## 2 结果

### 2.1 尼古丁处理后EMT相关分子标志物表达水平变化

提取各处理组细胞总蛋白，使用Western blot方法检测*E-cadherin*、*Vimentin*基因和内参基因β-actin蛋白表达。本研究观察到尼古丁诱导后EMT相关标志物表达水平发生明显变化。尼古丁处理肺癌细胞后Vimentin蛋白表达水平明显升高，不同浓度尼古丁处理组间Vimentin蛋白表达水平经F检验有统计学差异（*P* < 0.001）；两两比较：对照组与0.1 μmol/L尼古丁处理组间Vimentin蛋白表达水平比较有统计学差异（*P* < 0.05），对照组与1.0 μmol/L和10.0 μmol/L尼古丁处理组间Vimentin表达水平比较均有统计学差异（*P* < 0.001）（图 1），1.0 μmol/L与10.0 μmol/L尼古丁处理组间Vimentin表达水平比较有统计学差异（*P* < 0.001）（图 1）。尼古丁处理肺癌细胞后E-cadherin蛋白表达水平明显降低，不同浓度尼古丁处理组间E-cadherin蛋白表达水平经F检验有统计学差异（*P* < 0.001）；两两比较：对照组与0.1 μmol/L尼古丁处理组间E-cadherin蛋白表达水平比较有统计学差异（*P* < 0.05），对照组与1.0 μmol/L和10.0μmol/L尼古丁处理组间E-cadherin表达水平比较均有统计学差异（*P* < 0.001）（图 1），1.0 μmol/L与10.0 μmol/L尼古丁处理组间E-cadherin表达水平比较有统计学差异（*P* < 0.001）（图 1）。1 μmol/L尼古丁处理肺癌细胞24 h、48 h和72 h后Vimentin蛋白表达水平明显升高，不同时间尼古丁处理组间Vimentin蛋白表达水平经F检验有统计学差异（*P* < 0.001）；两两比较：对照组与24 h、48 h和72 h尼古丁处理组间Vimentin蛋白表达水平比较均有统计学差异（*P* < 0.001），24 h与48 h和72 h尼古丁处理组间Vimentin蛋白表达水平比较均有统计学差异（*P* < 0.001），48 h与72 h尼古丁处理组间Vimentin蛋白表达水平比较均有统计学差异（*P* < 0.001）（图 2）。1 μmol/L尼古丁处理肺癌细胞24 h、48 h和72 h后E-cadherin蛋白表达水平明显降低，不同时间尼古丁处理组间E-cadherin蛋白表达水平经F检验有统计学差异（*P* < 0.001）；两两比较：对照组与24 h、48 h和72 h尼古丁处理组间E-cadherin蛋白表达水平比较均有统计学差异（*P* < 0.001），24 h与48 h和72 h尼古丁处理组间E-cadherin蛋白表达水平比较均有统计学差异（*P* < 0.001），48 h与72 h尼古丁处理组间E-cadherin蛋白表达水平比较均有统计学差异（*P* < 0.001）（图 2）。尼古丁处理肺癌细胞株后Vimentin和E-cadherin蛋白表达水平变化具有时间和剂量依赖性（图 1，图 2）。

**1:**
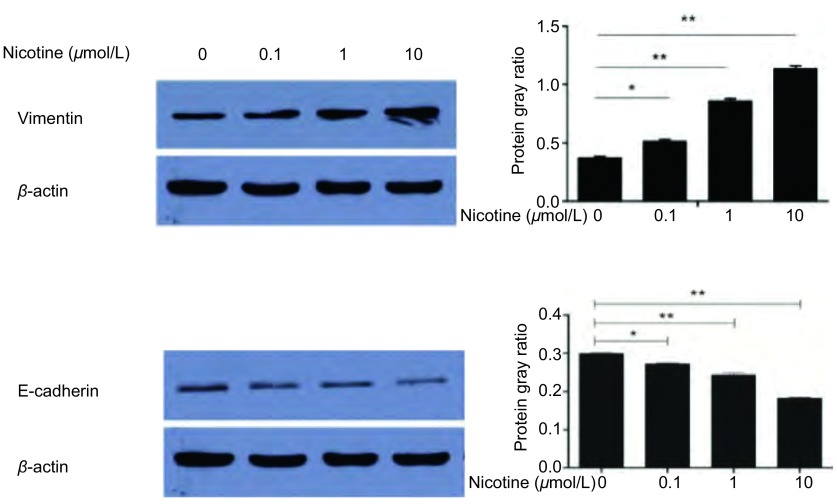
不同浓度尼古丁处理细胞24 h后EMT标志物变化及其灰度比值柱形图（^*^P < 0.05, ^**^P < 0.01） The expression levels of epithelial-mesenchymal transition (EMT) related protein makers in cells treated with different concentration of nicotine (0 *μ*mol/L, 0.1 *μ*mol/L, 1 *μ*mol/L, 10 *μ*mol/L) for 24 h and the bar chart of gray ratio value (^*^P < 0.05, ^**^P < 0.01)

**2:**
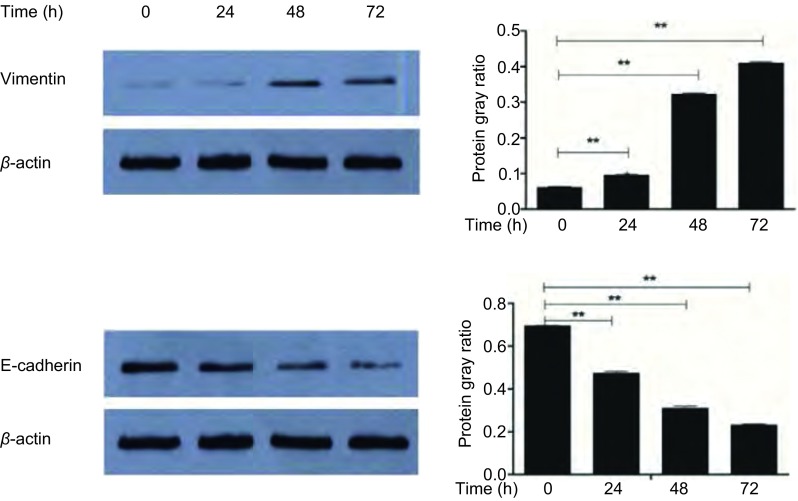
1 *μ*mol/L尼古丁处理细胞不同时间后EMT标志物的变化及其灰度比值柱形图（^**^P < 0.01） The expression levels of EMT related protein makers in lung cancer cells treated with 1 *μ*mol/L of nicotine for different times (0 h, 24 h, 48 h, 72 h) and the bar chart of gray ratio value (^**^P < 0.01)

### 2.2 尼古丁处理肺癌细胞后EMT相关分子标志物mRNA表达水平变化

应用Real-time PCR方法检测*E-cadherin*、*Vimentin*基因和内参基因*GAPDH*的表达。本实验采用2^-ΔΔct^值进行相对定量分析，本研究观察到：尼古丁处理肺癌细胞后Vimentin mRNA表达水平明显升高，不同浓度尼古丁处理组间Vimentin mRNA表达水平经F检验有统计学差异（*P* < 0.001）；两两比较：对照组与0.1 μmol/L、1.0 μmol/L和10.0 μmol/L尼古丁处理组间Vimentin mRNA表达水平比较均有统计学差异（*P* < 0.001）（图 3），0.1 μmol/L与1.0 μmol/L和10.0 μmol/L尼古丁处理组间Viment in mRNA表达水平比较有统计学差异（*P* < 0.001），1.0 μmol/L和10.0 μmol/L尼古丁处理组间Vimentin mRNA表达水平比较有统计学差异（*P* < 0.001）（图 3）。尼古丁处理肺癌细胞后E-cadherin mRNA表达水平明显降低，不同浓度尼古丁处理组间E-cadherin mRNA表达水平经F检验有统计学差异（*P* < 0.001）；两两比较：对照组与0.1 μmol/L、1.0 μmol/L和10.0 μmol/L尼古丁处理组间E-cadherin mRNA表达水平比较均有统计学差异（*P* < 0.001）（图 3），0.1 μmol/L与1.0 μmol/L和10.0 μmol/L尼古丁处理组间E-cadherin mRNA表达水平比较有统计学差异（*P* < 0.001），1.0 μmol/L与10.0 μmol/L尼古丁处理组间E-cadherin mRNA表达水平比较有统计学差异（*P* < 0.001）（图 3）。尼古丁处理肺癌细胞株后Vimentin和E-cadherin mRNA表达水平变化具有剂量依赖性（图 3）。

**3:**
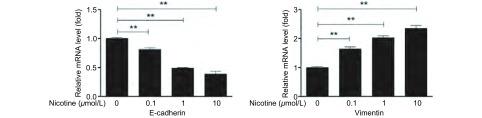
Real-time PCR检测不同浓度尼古丁处理肺癌细胞24 h后EMT相关标志物mRNA水平变化（^**^P < 0.01） The expression levels of EMT related makers in lung cancer cells treated with different concentration of nicotine (0 *μ*mol/L, 0.1 *μ*mol/L, 1 *μ*mol/L, 10 *μ*mol/L) for 24 h and the bar chart of gray ratio value (^**^P < 0.01)

### 2.3 尼古丁处理后肺癌细胞

β-catenin蛋白表达水平变化β-catenin蛋白主要位于细胞膜，与E-cadherin结合为复合体共同为介导细胞间粘附。当细胞发生EMT之后细胞膜E-cadherin表达减少，β-catenin与其分离，从而β-catenin进入细胞核。β-catenin蛋白表达的核转移间接证明EMT的发生。此次免疫荧光实验选用浓度为0 μmol/L和1 μmol/L的尼古丁两组进行比较。结果显示，1 μmol/L处理组细胞与对照组比较，β-catenin蛋白明显发生核转移，提示尼古丁处理的细胞发生EMT（图 4）。

**4:**
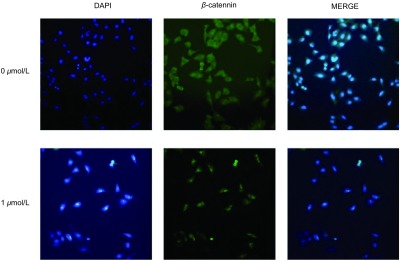
1 *μ*mol/L尼古丁处理肺癌细胞24 h后*β*-catenin蛋白发生核转移（×200） The *β*-catenin protein translocated to the cell nucleus after treating with 1 *μ*mol/L nicotine for 24 h (×200)

### 2.4 尼古丁处理后肺癌细胞迁移潜能变化

尼古丁处理肺癌细胞24 h、48 h后，进入划痕区域的细胞数量发生明显变化，划痕区域面积明显缩小。本研究观察到：0.1 μmol/L、1 μmol/L、10 μmol/L尼古丁处理组肺癌细胞进入划痕区域细胞数量较0 μmol/L组明显增多，差异具有统计学意义（*P*=0.001）。结果提示不同浓度尼古丁能够增强A549细胞迁移能力，并呈现浓度和时间相关性（图 5）。

**5:**
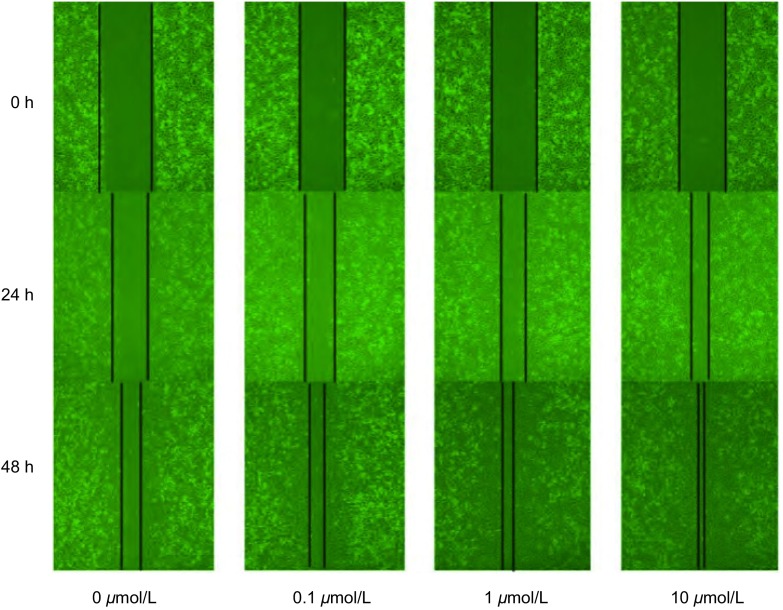
不同浓度尼古丁处理A549细胞24 h、48 h后迁移能力的变化（×40） The ability of migration was increased in cells treated with different concentration of nicotine (×40) (0 *μ*mol/L, 0.1 *μ*mol/L, 1 *μ*mol/L, 10 *μ*mol/L) for 24 h and 48 h

### 2.5 尼古丁处理后肺癌细胞体外侵袭潜能变化

Transwell侵袭实验设置尼古丁浓度为0 μmol/L、1 μmol/L两组进行实验。本研究观察到：1 μmol/L尼古丁处理组肺癌细胞穿过小室基底膜肺癌细胞数目（106±2）与0 μmol/L尼古丁处理组肺癌细胞穿过小室基底膜肺癌细胞数目（67±2）比较，明显增多，侵袭能力明显增强，差异具有统计学意义（*P*=0.001）（图 6）。

**6:**
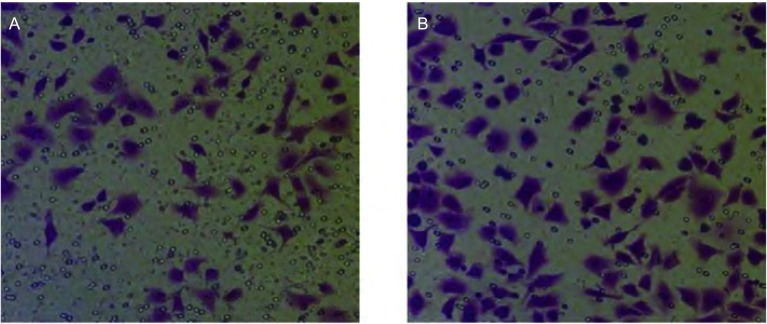
Transwell侵袭实验（×200）。A：对照组；B：尼古丁处理组。 The ability of invasion was increased in cancer cells treated with 1 *μ*mol/L nicotine (×200). A: Control group; B: Group of cells treated with 1 *μ*mol/L nicotine.

## 3 讨论

吸烟是引起包括肺癌在内的多种肿瘤相关性疾病死亡的首要危险因素之一^[3]^。已知香烟中存在着超过4, 000多种化学物质，其中至少有45种被认为可能是致癌物质^[15]^。大多数的香烟致癌物也是强诱变剂，能够与DNA形成加合物，从而激活原癌基因，而使抑癌基因失活^[16]^。虽然戒烟对肿瘤治疗相关并无太大影响，但是持续的吸烟可能降低治疗疗效甚至增加患者死亡率。癌症成功治疗后的戒烟却能降低新癌症的罹患风险率，非吸烟患者与以前吸烟和现吸烟患者比较有更长的生存期。已有的研究表明：尼古丁诱导的侵袭和EMT作用，可能是乳腺癌和肺癌进展的重要机制。尼古丁本身虽然没有强致癌作用，但它能促进癌细胞的增殖、侵袭、转移、血管生成以及抗细胞凋亡，还能诱导肺癌细胞产生EMT，这种作用主要发生在吸烟者尼古丁血浆浓度为10^-8^ M-10^-6^ M的患者^[17, 18]^。然而每吸完一支香烟，人体肺泡液中尼古丁含量便可高达6 μmol/L-60 μmol/L，而1 μmol/L的尼古丁浓度便可以诱导细胞抗凋亡，促进增殖及产生EMT^[7, 19]^。

EMT是一个高度保守的细胞程序化过程，上皮来源的细胞通过特定程序转化，使细胞上皮表型缺失并获得间充质细胞表型，固定的上皮细胞转化为能动的间充质细胞的过程。上皮细胞侵袭和迁移的能力主要是依赖于细胞骨架的重塑，从而导致细胞EMT，促进肿瘤获得转移能力^[20]^。EMT主要的特征是细胞E-钙粘蛋白分子表达的减少和波形蛋白的获得，并且同时获得间质细胞的形态学特征。E-钙粘蛋白属于钙依赖的粘附分子的钙粘蛋白家族，在细胞-细胞间相互作用的调控中起至关重要的作用^[21]^。作为保持表皮状态的关键因素，它表达降低和缺失与肿瘤不良预后紧密相关^[12]^。它与许多类型的肿瘤进展密切相关，并参与肿瘤的转移和治疗抵抗性^[22]^。重要的是除了参与肿瘤的转移，还与细胞的耐药性和肿瘤干细胞的产生有关。EMT是上皮来源的恶性肿瘤获得侵袭和迁移能力的重要生物学过程，上皮细胞通过恶性转化获得迁移能力，允许其侵入周围细胞基质，并通过血液和淋巴系统进行远处播散和转移。

本研究结果表明：香烟中重要的化合物质尼古丁处理后的肺癌细胞，无论是从蛋白水平还是mRNA水平均表现出明显的E-cadherin表达降低和Vimentin表达增强，提示尼古丁能够诱导肺癌细胞EMT。EMT是一个多步骤的过程，多种信号因子、转录因子和信号通路参与其中。参与的转录因子包括Snail家族（Snail1、Snail2/Slug和Snail3）、Twist、β-catenin、锌指结合蛋白（ZEB1和ZEB2）和TCF3/E47/E12等^[23, 24]^。尼古丁还可以通过Wnt/β-catenin信号通路诱导人呼吸道上皮细胞EMT，并能在体外环境下诱导人肺泡上皮细胞和肺癌A549细胞发生EMT^[25]^。肿瘤细胞核β-catenin蛋白的积累表明其同样经历了EMT，这些细胞E-钙粘蛋白的表达逐渐丧失，但间充质标志物表达却反向增高^[14]^。NSCLC晚期患者的E-钙粘蛋白基因沉默，表明E-钙粘蛋白表达下降参与肿瘤细胞的侵袭和转移^[26]^。相比之下，N-钙粘蛋白和波形蛋白表达的上调同样也与NSCLC的转移相关，而抑制其表达可以减弱NSCLC的增殖和侵袭能力^[27, 28]^。虽然大多数肺癌EMT相关标志物间质表型存在高表达，尤其是在鳞状细胞癌中，但无论E-钙粘蛋白的降低或是N-钙粘蛋白的过度表达都预示着NSCLC患者的预后不良^[29]^。本研究免疫荧光结果提示细胞膜表达的β-catenin蛋白，经尼古丁处理后发生了明显的细胞核聚集，即所谓的膜蛋白核转移，间接证明了尼古丁可以诱导肺癌细胞EMT。另外，划痕实验和Transwell侵袭实验，同样证明了肺癌细胞经尼古丁处理后，细胞迁移侵袭能力明显增强，这与肺癌细胞发生EMT紧密相关。

已有的研究证明：手术目前仍然是NSCLC的主要治疗手段，然而治疗后的复发和转移，仍然是肺癌治疗失败的主要因素。尼古丁作为烟草中重要化学物质，与NSCLC的发生紧密相关，它能够诱导肺癌细胞EMT，还能促进肺癌干细胞样细胞增多。肿瘤细胞获得EMT后，其迁移和侵袭的能力明显增强，并获得干细胞样特征和细胞耐药性，通过抑制EMT，可以降低肺癌细胞的侵袭和转移能力，阻断EMT表型和信号通路，还可以增强化疗敏感性^[27, 28, 30, 31]^。考虑EMT在癌症的发展过程中的重要作用，针对涉及EMT而提供的治疗策略，可能对防止肿瘤的复发转移、耐药性产生至关重要。因此，研究和开发肺癌EMT相关的分子靶点及分子靶点小分子药物，对改善NSCLC吸烟的患者的临床治疗效果及预后具有重要的理论意义和广阔的应用前景。
